# *Legacy for Children*^*TM*^: a pair of randomized controlled trials of a public health model to improve developmental outcomes among children in poverty

**DOI:** 10.1186/1471-2458-12-691

**Published:** 2012-08-23

**Authors:** Ruth Perou, Marc N Elliott, Susanna N Visser, Angelika H Claussen, Keith G Scott, Leila H Beckwith, Judy Howard, Lynne F Katz, D Camille Smith

**Affiliations:** 1 Centers for Disease Control and Prevention, 1600 Clifton Road, Atlanta, GA, 30333, USA; 2 RAND, 1776 Main Street, P.O. Box 2138, Santa Monica, CA, 90407, USA; 3 University of Miami, Linda Ray Intervention Center, 750 NW 15 St, Miami, FL, 33136, USA; 4 University of California at Los Angeles, 300 UCLA Medical Plaza, Suite 3300, Los Angeles, CA, 90095, USA

## Abstract

**Background:**

One in five Americans under age 18 lives in a family below the Federal poverty threshold. These more than 15 million children are at increased risk of a wide variety of adverse long-term health and developmental outcomes. The early years of life are critical to short- and long-term health and well-being. The *Legacy for Children*^*TM*^ model was developed in response to this need and marries the perspectives of epidemiology and public health to developmental psychology theory in order to better address the needs of children at environmental risk for poor developmental outcomes.

**Methods/design:**

The *Legacy for Children*^*TM*^ group-based parenting intervention model was evaluated as a pair of randomized controlled trials among low-income families in Miami and Los Angeles. The study was designed to allow for site-stratified analysis in order to evaluate each model implementation separately. Evaluation domains include comprehensive assessments of family, maternal, and child characteristics, process outcomes, and prospective programmatic cost. Data collection began prenatally or at birth and continues into school-age.

**Discussion:**

The societal costs of poor developmental outcomes are substantial. A concerted effort from multiple sectors and disciplines, including public health, is necessary to address these societal concerns. *Legacy* uses a public health model to engage parents and promote overall child well-being in families in poverty through rigorous evaluation methodologies and evidence-based intervention strategies. This study collects rich and modular information on maternal and child outcomes, process, and cost that will enable a detailed understanding of how *Legacy* works, how it can be refined and improved, and how it can be translated and disseminated. Taken together, these results will inform public policy and help to address issues of health disparities among at-risk populations.

**Trial registration:**

NCT00164697

## Background

One in five Americans under age 18 lives in a family below the federal poverty threshold [1]. These more than 15 million children are at increased risk for a wide variety of adverse long-term health and developmental outcomes [2-5]. In particular, such children are at increased risk for poor developmental outcomes, including developmental delays and disabilities, special education placement, and academic failures, as well as poor health outcomes [6-9].

Over several decades of research, the roles of early experience in determining lifelong learning, emotional and physical well-being, social attainment, and presence or absence of chronic disease have been documented [10-14]. Because many of the behaviors and health conditions associated with adult morbidities and mortality (e.g., obesity, smoking, and substance abuse) begin in early childhood, this life stage represents a critical time for intervention [12,15]. The strong association between the nature of early experience and short and long-term health and well-being make early childhood environmental risk associated with poverty a preventable public health concern.

Between 1994 and 1998, the Centers for Disease Control and Prevention (CDC) began to engage in and fund activities that sought to better understand interventions to help mediate adverse health outcomes for children and families that face vulnerabilities. The impetus was a growing body of evidence suggesting differences in cognitive outcomes by race/ethnicity, potentially mediated by poverty [13]. A review of the substantial early intervention literature at the time revealed several important findings. The majority of studies that demonstrated program effects were center-based, with direct provision of the intervention to children [16-21]. Despite a wealth of research that focused on parent education, limited work had been done to promote healthy development of children at elevated risk for poor developmental outcomes by focusing on parents as the agents of change [22,23]. Group-based interventions with parents and children were a promising and potentially less resource-intensive early intervention format [24-26] but evaluation data were limited [17]. It was also clear that intervention effectiveness could significantly vary across sites and even within samples [27,28]. Thus, it was critical to allow for implementation flexibility while measuring model fidelity across sites via a process evaluation. Finally, a common challenge across existing early intervention outcome evaluations was related to measurement and statistical power [13,17], which pointed to the need to collect a wide variety of child outcomes on a large enough sample to allow for the comparison of intervention effects both within and across implementation sites over time.

Previous interventions were often focused around a specific domain of child outcome, e.g., intellectual development, child behavior problems. From a public health perspective, all of the major domains of child outcomes are important, as intellectual, communicative, social/emotional, and behavioral outcomes are connected to long term health and functioning (e.g., [13,29]). Furthermore, early interventions that were aimed at one domain of outcome ended up benefiting other domains. For example, the Perry Preschool program was intended to improve IQ, but demonstrated long term benefits in socially adaptive behaviors, such as decreased delinquency and increased school and economic success [30]. Thus, *Legacy for Children*^*TM*^ specifically addressed a broad range of child outcomes.

There was a need to build upon research to develop and rigorously test an early intervention model that targeted parents as agents of change through the use of a potentially more efficient group-based intervention format that allowed for adaptation within implementation sites. The study would require sufficient sample sizes to allow for statistical power to detect group differences in both child outcome and mediating factors. In addition to collecting outcome data related to the development of participant children, detailed process data were also needed in order to evaluate model provision, adherence, and intervention paths. The *Legacy *model was developed in response to this need and marries the perspectives of epidemiology and public health to developmental psychology theory in order to better address the needs of children at environmental risk for poor developmental outcomes.

*Legacy* is a public health model evaluated in a controlled setting but intended to be applicable at the community level. The core elements of the intervention provide structure but allows for adaptation to the needs of communities. Effectiveness testing was conducted using a randomized controlled evaluation protocol that incorporated a full-length pilot study, measurement of a wide variety of maternal outcomes, child outcomes, and intervention process variables. This paper describes the *Legacy* model and the methods used to test its effectiveness in two research sites between 2001 and 2009. Notably, the following description of the *Legacy* model is a conceptual one and should not be confused with the description of a “model program.” Rather, model programs result from using the model to develop a fully functional intervention program, such as the two individual programs that resulted from model applications, both of which will be described later in this manuscript.

### The *Legacy* philosophy

Parenting is one of the most important tasks of the family, and one of the most challenging, yet gratifying, roles in our society. Parents are the key to the provision of safe, nurturing, and positive learning environments for children as they grow and mature. Past research indicates that the personal characteristics that present in successful children are consistently correlated with parental influences [13,31]. Children who face risk factors such as poverty are more likely to overcome these challenges when their parents are involved and invested in providing a safe, strong base of support [32-34]. Children who do well in life despite being born into less than ideal circumstances often cite their parents as the factor that made the difference [35].

The focus for *Legacy* was the contention that families living in isolation from the larger social context of their communities face greater challenges in raising their children. This particularly holds true for families living in disadvantaged communities where barriers to establishing supportive relationships with others and the adverse consequences to child development are great. Support for parents outside the family is important to ameliorating the stress and barriers to effective parenting. Convincing evidence at the time supported the contention that extra-familial, instrumental supports and supportive relationships contribute significantly to better child developmental outcomes [36-42]. Social support provided to parents by social networks had been shown to be effective in reducing parenting stress including specific types of social support such as assistance, information, empathy, and understanding [43,44]. These types of support can have a direct effect on maternal regulation of child behavior, with the strongest effects for families living under stressful conditions [41,43]. Moreover, mothers who indicate greater social support, both received and perceived, have been shown to have more sensitive and positive interactions with their children [45-49]. Thus, a group approach to parenting intervention, the development of a sense of community among mothers, as well as the interactions with the group leaders to promote positive changes in their parenting behavior [50] was identified as the model to be developed. In addition, this approach would potentially be more cost effective than center-based intervention for children.

*Legacy* was designed to promote optimal child outcomes by enhancing the mothers’ feelings of self-efficacy in their parenting tasks. Perceived self-efficacy is the belief that one has the power to produce effects by one's actions [51]. Maternal self-efficacy helps mothers assess their behaviors and achieve behaviors that will positively influence the development of their children [52,53]. *Legacy* promotes the belief that mothers can successfully parent, regardless of their current life circumstances, if given the opportunity to improve their parenting knowledge and their parenting behaviors while also acknowledging that it takes time and is a dynamic process. The *Legacy* philosophy includes the belief that mothers do a better job of adopting and maintaining behaviors that enhance their child’s development if they receive ongoing support for these behaviors from a peer group and are able to develop a sense of belonging to a community larger than themselves.

### Legacy assumptions, goals, and core intervention activities

#### Development of the intervention

**Figure 1:**
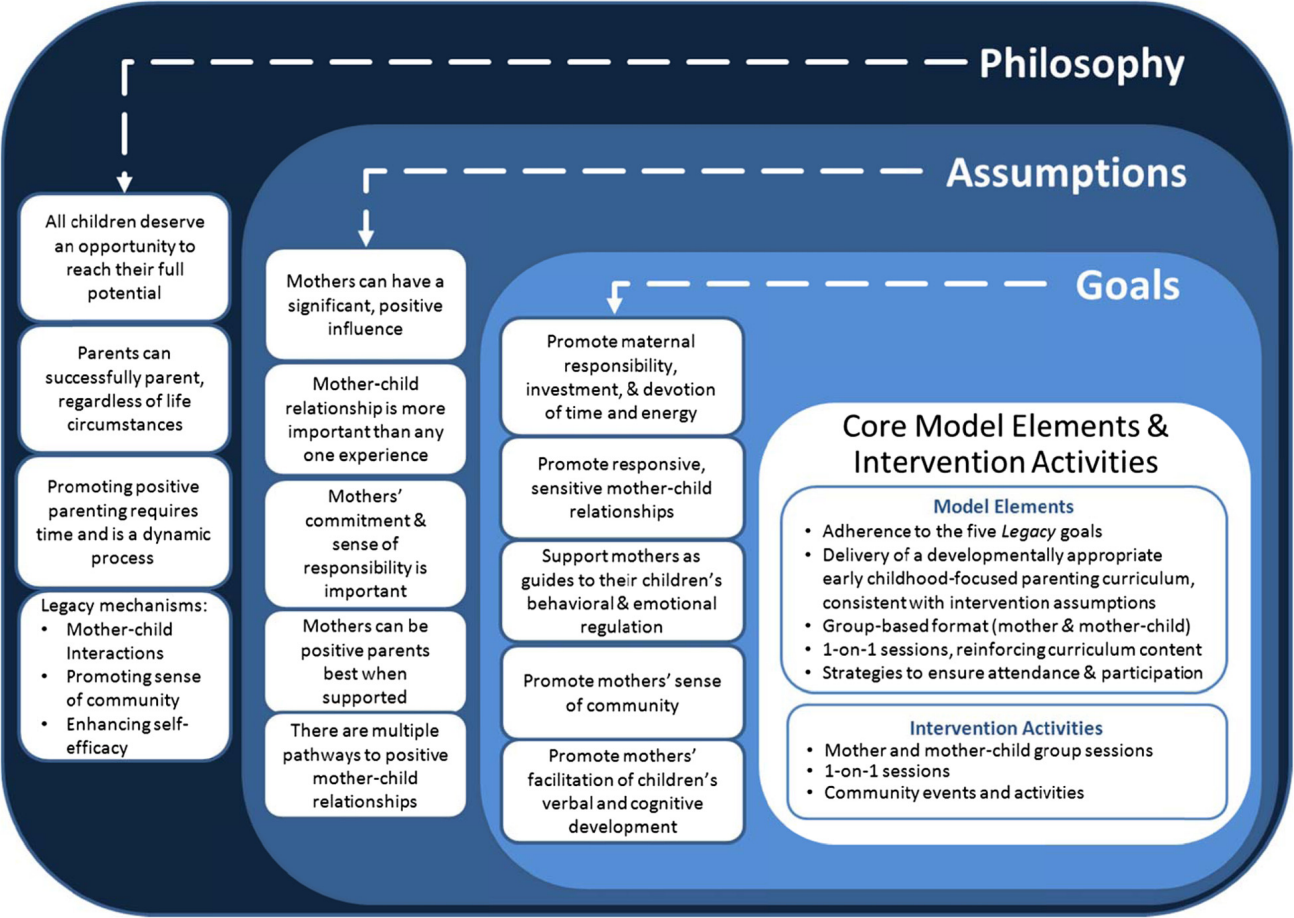
**Legacy for Children**^**TM**^**conceptual model.**

CDC conducted multiple expert workgroups to help lay the theoretical foundation for the intervention and intervention goals. Additionally, in collaboration with the Project Coordinating Center (PCC; RTI International) and with consultants, extensive literature reviews on child development, parenting, early childhood interventions, and sense of community were conducted, as well as a comprehensive environmental scan of early childhood programs. The PCC also developed and maintained an Intervention Resource Library containing early childhood curricula and other relevant resources for program implementation. The resulting conceptual model is described below and is visually depicted in Figure 1. Although the *Legacy* philosophy was grounded in parenting literature, the knowledge base for mother-child relationships was more fully developed at the time. The *Legacy* model was ultimately developed and operationalized with mothers in mind.

#### Model assumptions

Complementing the philosophy of *Legacy* is a set of five model assumptions (A1-A5) undergirding the entire intervention. These assumptions were established prior to the development of each implementation site’s curriculum. See Figure 1.

A1. Mothers can have a significant positive influence on their children’s short-term and long-term development.

A2. The quality of the mother-child relationship is more important than any particular experience mothers provide to their children.

A3. An important factor in parenting is the mothers’ commitment and sense of responsibility for making deliberate and thoughtful choices in furthering their children’s development.

A4. Mothers can develop and sustain positive parenting best when they experience social support, such as that from other mothers who share a sense of parental responsibility.

A5. There are multiple pathways to positive mother-child relationships.

#### Study goals

The philosophy and model assumptions were the basis from which the specific *Legacy* intervention goals were developed (G1-G5). The goals of the intervention were to:

G1. Promote maternal responsibility, maternal investment, and maternal devotion of time and energy.

G2. Promote responsive, sensitive mother-child relationships.

G3. Support mothers as guides to their children’s behavioral and emotional regulation.

G4. Promote mothers’ sense of community.

G5. Promote each mother’s facilitation of their children’s verbal and cognitive development.

#### Core model elements and intervention activities

Taken together, the *Legacy* model has a core set of parameters or design elements within which an intervention may be adapted. However, in order to maintain fidelity with the *Legacy* model The core elements that are required for *Legacy* model adherence include: 1) Adherence to the five *Legacy* goals; 2) Delivery of a developmentally appropriate early childhood-focused parenting curriculum consistent with intervention assumptions; 3) Group-based format (mother and mother-child); 4) One-on-one sessions that reinforced curriculum content; 5) Strategies to ensure attendance and participation (e.g., child care provisions, transportation).

Notably, a specific curriculum was intentionally omitted from the *Legacy* model, as at the time of conceptualization no one parent education curriculum appeared to confer greater benefit than others. Thus, implementation sites were asked to develop their own developmentally appropriate early intervention curriculum that was consistent with the unique philosophy, assumptions, and goals of the *Legacy* model while being responsive to the specific challenges and opportunities of families in their communities.

The *Legacy* model requires the infusion of the *Legacy* goals throughout the intervention. To provide structure for the intervention, three core intervention activities were identified (C1-C3). As with the curriculum, it was not specified how the core activities needed to be structured; sites were able to use different formats as long as they included these activities:

C1. Mother and mother-child group sessions. Weekly group meetings were the format through which the intervention concepts were introduced. Mother-only meetings allowed for activities focused on building a sense of community among mothers, and for in-depth discussions of the information that was presented. In the mother-child group meetings, mothers practiced concepts learned in the group meetings.

C2. One-on-one sessions. Periodic one-on-one sessions between the mothers and group leaders were designed to reinforce concepts presented at the group meetings and to address individual parenting concerns. These sessions initially took the form of home visits but were also delivered by pulling mother-child dyads aside within the group setting.

C3. Community events and activities. These occasional special events were designed to promote group cohesion among mothers and to reinforce and expand on building a sense of community. This component could include activities such as birthday parties, group outings to the park, library, or community events; and group dining. The mothers had some responsibility in planning these events. Optional activities included encouraging maternal involvement in the larger community (e.g., church/religious activities, school involvement, and other community organizations) and promoting individual contributions to the community.

#### Development of the *Legacy* curricula

Two sites were awarded *Legacy* implementation contracts, based on competitive review. The scope of the contracts included the development and delivery of an intervention curriculum that was consistent with the *Legacy* model. The site-specific interventions reflected the core assumption that parents were more likely to adopt and continue with positive parenting behaviors if they developed a sense of belonging to a community of like-minded peers—other parents with shared values and a commitment to parenting. At both study sites (the University of Miami, or UM, and the University of California at Los Angeles, or UCLA), the core component of the intervention took the form of weekly parent group sessions, supplemented with additional social activities to build a sense of community among participants. These group meetings were facilitated by professionals with child development expertise. The parent group sessions, which met regularly throughout the early years of childhood, were designed to provide emotional, practical, and informational support to mothers. Each site developed its own intervention approach and specific curriculum to reflect the conceptual framework and goals of *Legacy* while keeping in mind the unique characteristics of the participants and the community. Both UM and UCLA also included special events including field trips, birthday celebrations, and other festive social gatherings; these events were aimed at fostering a sense of community among the mothers, maintaining interest and investment in the study, and extending opportunities for mothers to discuss topics covered in the group meetings.

#### Site implementations of the *Legacy* model

The *Legacy* intervention was longer than typical group-based parenting programs at the time (3 years in UCLA; 5 years in UM) with the intention of optimizing the opportunity for relationships to take root and grow. The guiding principle of the *Legacy* approach was that the group context affords a contained, small-community venue for guided learning, mutual aid, reinforcement of new ideas and skills, and an expanded social network. It was anticipated that the participants would grow to value the small parenting group context as a source of support, information and friendship encouraging them to continue to seek out those sources of support after transitioning from *Legacy*. Both study sites developed their own curricula reflecting the above shared core components and intervention goals. Each site developed a critical factors matrix that described their goals, objectives and curricular sessions. UCLA and UM had similar pre-implementation training that included an overview of the *Legacy* philosophy, how to be an effective group leader and the importance of and how to promote self-efficacy. CDC requested that the sites develop their curriculum to adhere to the core components and intervention goals and ensured this adherence through qualitative processes. Each site was closely monitored to ensure fidelity by CDC and the PCC. The sites were different in terms of their start time and length, format and structure, incentives, education and training of the staff. The provision of the intervention was also different because they took into account the local community, demographics and cultural aspects of the participants. Each site carefully considered who should deliver the intervention, where the intervention should be administered, what the content should be and how long it should be based on their unique set of circumstances.

#### *Legacy* UCLA

The UCLA approach was built around three principles: 1) intervene when mothers are uncertain and motivated to learn needed skills; 2) training in parenting behavioral skills is effective; and 3) time-limited interventions help prevent participants’ burn out and promote learning.

The UCLA intervention began when the participants were approximately 7 months pregnant and continued until the child was 3 years old. At UCLA, the intervention design centered on weekly 1-hour parent group sessions delivered in 10 week blocks followed by a break of 4 to 6 weeks. These sessions were led by a professionally trained group leader, with session types alternating between mother-child sessions and mother-only sessions. The purpose of the mother-child sessions was to support the development of positive, supportive relationships between each mother and her child, by practicing new skills, observing how other mothers interacted with their child, and by the group leader modeling interactive behaviors and providing guidance if needed. For the mother-only sessions, participants also engaged in what was called FUN Club (Family Unity Network Club). FUN club was designed to provide mothers with additional unstructured time to socialize and to plan and do crafts or other activities together, to support their sense of community. In addition to the parent group sessions, the intervention design included two other components: (1) periodic one-on-one visits to the home by the group leader to provide individual attention and to review topics from the group sessions; and (2) community-building events and activities, such as field trips or birthday celebrations.

#### *Legacy* UM

The UM approach was built around “reality-based parenting.” Miami developed an approach that would impart developmentally appropriate practices and child development information into an interactive, situational-based framework, garnered from previous interactive experiences with their culturally diverse families in the Miami community.

The parent group sessions began approximately 2 to 3 months postpartum, were held weekly with only short breaks for holidays, and continued until the child was 5 years of age. Each 90-minute session comprised three segments facilitated by a group leader with professional training in early childhood development: (1) a “Building Sense of Community” (BSC) portion, which provided unstructured time to allow for parental sharing of concerns and for fostering group cohesion, peer support, and mutual aid; (2) a “Main Session Topic” (MST) portion, in which a parenting topic was presented in a hands-on, interactive manner (e.g., with games and activities); and (3) “Parent child Together Time” (PCTT) in years 1 to 4 and “Creative Learning Activities for Time Together” (CLATT) in year 5. The PCTT portion of the parent group session was one-on-one time for the group leader to support and coach mothers during mother-child interaction activities. The primary objective of PCTT was to support the development of positive, supportive relationships between each mother and her child. CLATT was designed to support mothers in preparing their children for kindergarten by showing them games and activities (e.g., that were literacy, numeracy, sorting/classification oriented) to play at home together. The length of time spent on each portion of the session was adjusted to adapt to group needs. For example, as children grew older and enrolled in child care or preschool, the group leader would extend the BSC and MST time. On-site child care was provided during the mother-only portions of each session (i.e., community-building time and parenting topic discussions or activities).

In Miami, the final parent group meeting of every month was designated a “party” session. During these sessions, mothers engaged in a planned hands-on activity designed to encourage relaxed social interaction. The party sessions also included a special snack. Depending on interest and scheduling availability, field trips to community locations (e.g., fire station, children’s museum) were arranged each year for mothers, target children, and their siblings. The UM intervention design included one-on-one sessions with group leader, to reinforce concepts being covered in the group meetings. The one-on-one component included two sessions during the first year (the first made approximately 1 week before the first parent group session), with one session in each of the subsequent years, a total of six one-on-one sessions during the 5 years of the intervention. Originally these sessions were intended to be conducted at the mother’s home, however, many mothers were not comfortable with being visited at home, so one-on-one sessions were switched to be conducted at the site location in conjunction with group meetings.

### Study hypotheses

The ultimate goal for *Legacy* was to improve outcomes for children along a broad spectrum of developmental domains. Therefore, the primary research question was: Do the children of mothers in the intervention groups achieve better developmental outcomes than do the children of mothers in their respective comparison groups?

A corollary set of questions asked in research of this type involves measuring corollary effects associated with the intervention. Thus, supplementing the main research question were five related questions.

Q1. Do mothers become engaged in the intervention program?

Q2. Do mothers adopt parenting behaviors more likely to foster child development?

Q3. Do the site adaptations of the model have similar or different effects on the measured outcome variables?

Q4. Do mothers in the intervention program develop a greater sense of community?

Q5. What are the costs of delivering the intervention and do the potential long-term benefits outweigh the costs?

Of these, Q2-5 involve comparing intervention and comparison groups, whereas Q1 will involve an analysis of intervention data only.

## Methods/design

### Evaluation aims

Based on the initial review of early intervention research, internal and external expert input, and the development of the *Legacy* model, five related study aims were identified:

Aim 1: Document the implementation of the *Legacy* intervention and evaluate intervention fidelity.

Aim 2: Determine the relationship between self-efficacy and sense of the community and positive maternal-child interaction among the *Legacy* intervention mothers versus the comparison group mothers.

Aim 3: Evaluate the long-term goals of the intervention by examining the developmental outcomes of the children of the *Legacy* intervention mothers versus the comparison group mothers, in the domains of cognitive, language, socio-emotional, and behavioral development.

Aim 4: Understand how mothers responded to the intervention and which factors affected the quality of intervention each mother received.

Aim 5: Determine the costs associated with the group-based intervention in order to calculate overall costs and, if the intervention yielded significant effects, cost-benefit and cost-effectiveness indices.

**Figure 2:**
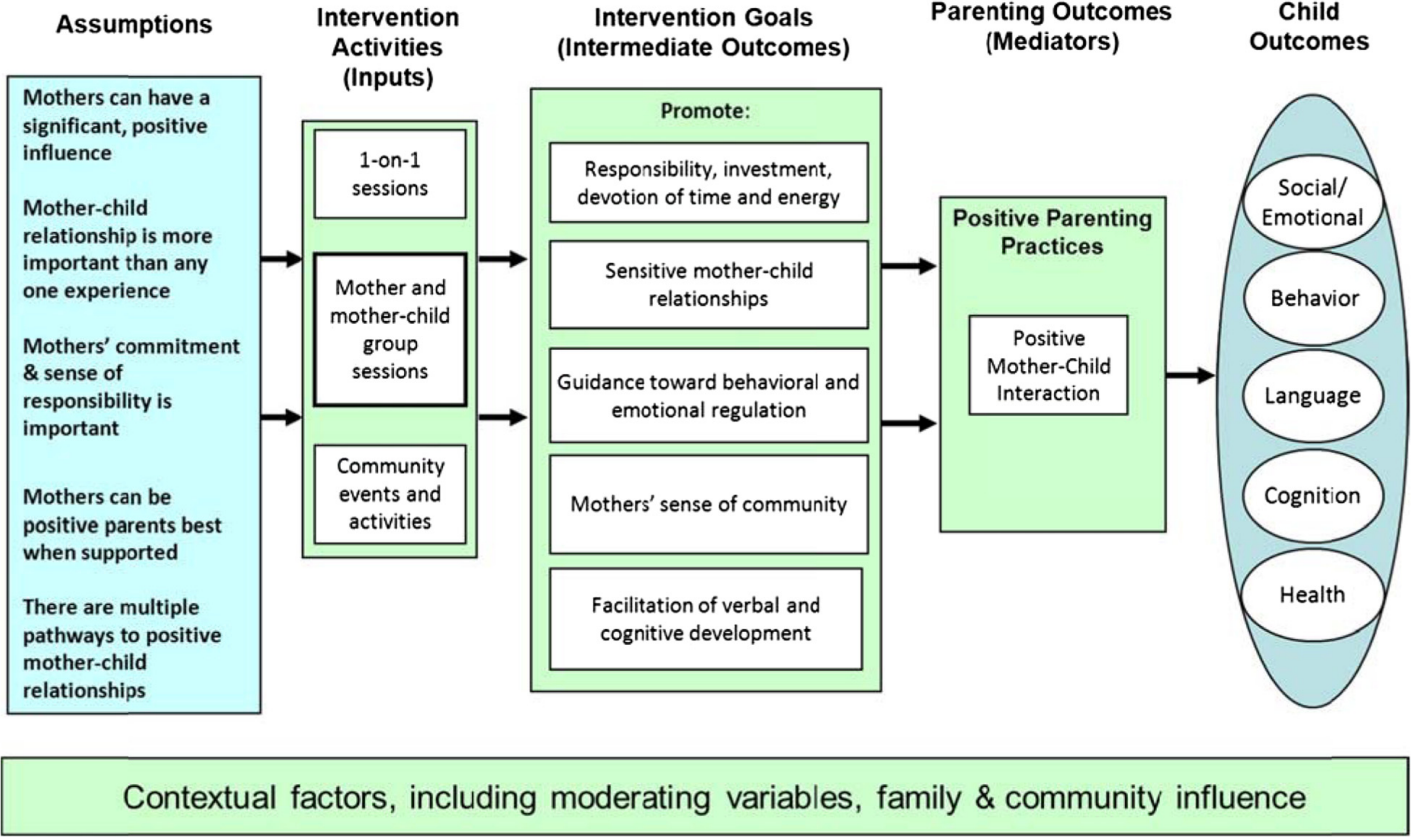
**Legacy for Children**^**TM**^** logic model.**

These aims were executed, in concert with the Legacy Logic Model (Figure 2).

### Site selection

As stated earlier, two implementation sites were selected based on a competitive award process: University of Miami and University of California at Los Angeles. In order to increase the likelihood of a objective program evaluation, the randomization process and the evaluation data were coordinated by the independent PCC, including collection and processing of all assessment data as well as those process data that were not based on direct observation by the intervention staff.

### Study design

#### Randomized controlled clinical trial

The evaluation design that was selected to test the impact of the *Legacy* model was a pair of randomized controlled trials, which was registered as such with ClinicalTrials.gov (Identifier: NCT00164697). Participants randomly assigned to the intervention group received intervention and contact with intervention staff on a regular basis, as described in the Site implementation section, above. The children in the comparison groups did not receive any core components of the *Legacy* intervention model but participated in the same schedule of developmental assessments. Families in this “usual care” comparison group were not prevented from utilizing any service that would otherwise be available to them, even if the service was similar to the services received in the intervention arm of the study. It was anticipated that families in the comparison group received some community services for which they were eligible, e.g. primary health care services, immunizations, and other early intervention programs. Families in both the comparison and intervention arms of the study were referred if the child scored in the risk range on standardized assessments (e.g., 1.5 standard deviations below the mean on IQ scores). Therefore, the comparison group represented a mild intervention group and tests of intervention effectiveness are conservative.

The intervention and evaluation procedures were pilot tested at each implementation site using the full-length pilot phase with 60 dyads at each site to test recruitment and retention procedures, obtain feedback on intervention content and delivery, and refine and test the multi-phasic assessment battery for process, outcome, and cost data. Although such pilot efforts have been shown to be extremely helpful in launching complicated social science investigations and are recommended as part of intervention development [54], they have not typically been utilized in early intervention research due to limited resources [55]. The pilot study data were not used to assess the effectiveness of the intervention but to refine and adapt the procedures and methods to the participant population.

### Target population

#### Inclusion criteria

The cross-site inclusion criteria had several components -- one that defined the target population and two that were logistically necessary. Pilot data were used to examine recruitment procedures and feasibility of recruitment criteria. The first criterion was environmental risk for poor developmental child outcomes, which was operationalized differently by the two sites. Families were selected based on environmental risk rather than medical or biological risk. Therefore, both intervention sites recruited from well-care settings and excluded mothers reporting mental health or substance abuse problems.

The second two criteria were factors that were required to facilitate intervention provision. The first of these logistical necessities was comfort speaking and understanding conversational English, given that the intervention was conducted in English, the assessment instruments were largely available only in English, and it was not feasible at either site to create groups that could be conducted in other languages. A second such criterion related to custody and age; because the intervention included attendance by mothers and their children, only mothers who had custody of their children and who were of age to consent to their own participation (18) were included in the study. Mothers who lost custody during the course of the intervention were no longer eligible to participate.

In addition to the aforementioned cross-site *Legacy* inclusion criteria, each site elected to impose additional site-specific criteria. The original eligibility criteria for the UCLA project further restricted eligibility to women who lived within 10 miles of UCLA, planned to stay in the LA area for 3 years, and received their prenatal and well-baby medical care from the UCLA Medi-Cal Health Maintenance Organization (which is a public health insurance program which provides needed health care services for low-income individuals). For feasibility issues related to intervention and assessment provision, mothers who had more than 2 children (including the target child), or were expecting a multiple birth, if known at the time of recruitment (26 weeks), were not eligible.

The original eligibility criteria for the UM project further restricted eligibility to mothers who lived within a 50-minute drive of at least one of the two community intervention sites, who gave birth at Jackson Memorial Hospital (the main teaching hospital in Miami-Dade County), and who planned to stay in the area for 3 years. Similar to UCLA and for feasibility issues, mothers who had multiple births or had more than two other children or children older than 4 years were excluded. UM operationalized poverty by including mothers who lived in specific zip codes selected because of low performing schools and schools with a high percentage of free lunch eligibility, as well as including only mothers who reported less than 12 years of maternal education.

#### Modification of main study inclusion criteria as a function of pilot findings

Several important lessons were learned from the pilot study recruitment process. First, the formal screening procedures used to identify maternal mental health and substance use problems were difficult and unreliable to implement in the pilot, so formal screening for these issues was dropped. However, clinic and hospital staff assisted *Legacy* staff by referring only mothers with no known medical risk factors, such as maternal mental health problems, substance use problems, and high-risk pregnancies. Second, the pilot study recruitment proceeded very slowly, particularly in Miami. The majority of mothers approached at that site could not be included because they were ineligible, rather than not being interested. Specifically, 249 of 316 mothers (79%) approached in Miami for the pilot were ineligible. Only 7 eligible mothers (10%) choose not to participate. In comparison, of 106 LA mothers screened for the pilot, 31 (29%) were ineligible, and 12 eligible mothers (16%) chose not to participate. The low eligibility rate of the Miami mothers, if it continued into the main study, would limit the generalizability of the results. Preliminary analyses also revealed that the mothers included in the Miami pilot were significantly less resourced and were at greater demographic risk than the LA mothers (61% vs. 40% household income < $20,000; 13% vs. 22% married, 71% vs. 43% living in neighborhoods with high unemployment), probably due to differences in how poverty was operationalized at the sites. Therefore, inclusion criteria were broadened and aligned across the two sites for the main study.

Specific changes to the project inclusion criteria included dropping the restriction on number or age of siblings at both sites. The remaining changes were implemented in Miami alone. Specifically, the criterion regarding maternal education and recruiting from zip codes with low performing and low-income schools was dropped for Miami, in favor of recruiting mothers who were eligible for state programs such as Medicaid, food stamps, or TANF. Ultimately, the final UM main study eligibility criteria included mothers who currently lived within 50 minutes of one of the community intervention sites, gave birth at Jackson Memorial Hospital or Jackson North Maternity Center, expected to stay within the catchment area for three years, and had a household income below 200% of the poverty level as indicated by Medicaid, food stamps, or TANF eligibility. In addition, to better align the Miami post-natal participant group with that of LA, the UM main study participants were required to report having had at least one prenatal care visit.

### Sample size and power

A key consideration for this intervention study was to determine the sample size required to detect meaningful effects of the intervention. Sample size calculations were conducted before study initiation to guide recruitment, informed by pilot study attrition rates and informed by the literature and knowledge of the time. During the study planning period, staff did not know how many sites would be funded to implement the *Legacy* model. Therefore, sample size calculations were conducted to allow for sufficient power to detect meaningful effects at each funded site.

Although the focus of the intervention model is on overall child development, the sample size estimation was based on an age-appropriate cognitive measure at the end of the intervention. The rationale for this follows. First, child cognition is positively correlated with other child development outcomes in the early years of life. Second, it has been shown to be a good predictor of later child outcomes. Third, the majority of early intervention studies have used child cognition as an outcome measure [56]. Fourth, as mentioned earlier, most policy makers place an emphasis on cognitive outcomes. As such, there is more literature available on the expected potential effect size for cognition than for other child development domains.

The key element in the statistical estimation of sample size was specification of the magnitude of the effect size (i.e., the absolute difference in group means divided by the assumed common standard deviation) that we want to be able to detect. Findings from previous parenting education programs have found small effect sizes (< 0.25) with respect to child development outcomes [56-58]. However, the proposed model varies from the traditional parenting education programs in its focus. The focus of this intervention is on the role of parents in the development of their children and presumes that parents can successfully address this role independently of their own personal circumstances or external stressors in their lives. Findings from early intervention efficacy randomized controlled trials (RCT) of children whose parents were from low socioeconomic strata have shown up to a full standard deviation difference in mean cognitive level between the intervention and comparison groups [16,59-61].

We chose an effect size of 0.50 for the age-appropriate cognitive measure in this study. Several considerations led to this choice. First, *Legacy* might be considered less intensive, with reference to the group leader working directly with the child, than some previous early intervention studies. A second consideration is that although previous parenting interventions found small effect sizes, this intervention will be different in intensity than these programs. The intervention will most likely be more intensive with reference to parent–child interaction than previous parenting interventions. A third consideration was the unknown level of noncompliance that would be encounter in the intervention group. In the Infant Health and Development Program, the level of program participation was found to be strongly related to the estimated effect size, with the observed difference in mean Stanford-Binet scores between the intervention subgroup with the lowest participation rate and the comparison group being about .25 standard deviations [62]. The level of intensity, with reference to previous early intervention and parenting programs, and low participation in the intervention by intervention families have the potential to affect the observed effect size.

Another important element to take into consideration when calculating sample size is the participant loss rate. The literature indicated a wide range, 7% to 70%, of subject loss rates for various early intervention programs [56,63]. The 7% loss rate was for a highly structured study where extraordinary measures were taken to minimize subject drop out [63]. It was highly unlikely that similar measures could be be employed in this study. Most studies have shown a 30% to 40% loss rate (37% [64]). Even with a conservative, anticipated loss rate of 50% by age five, the study would still have a power of 0.86 for detecting an effect size of 0.50 at each site. It should be noted that this sample size (120) refers to the total number of children (intervention and comparison) for whom data on an age-appropriate cognitive measure will be available at the end of the assessment period (age five). The computations assume a one-sided test at the alpha = 0.05 level. A one-sided test was chosen since the available literature on early intervention gives no indication of a detrimental effect on a child’s development.

Based on data from the pilot study, we had a 50 - 70% attendance in the mothers’ groups. In order to ensure a practical group size (approximately 7–10 per group) we decided to recruit 15 intervention mothers per group for the main study, resulting in a recruitment ratio of 15 intervention mothers to 10 comparison mothers. Because we experienced approximately an 80% assessment compliance rate, we did not increase the number of comparison mothers. The pilot study also suggested that the rate of fetal loss that should be expected among the prenatally recruited LA participant group was 5%. Therefore, the final main study sample included 300 Miami and 315 LA participants who were randomized in a ratio of 3 intervention: 2 comparison, resulting in 180 intervention and 120 comparison participants in Miami, and 190 intervention and 125 comparison participants in LA. Post-randomization, 9 participants became ineligible due to fetal loss (*n* = 3) and administrative recruiting errors (*n* = 6).

### Recruitment and randomization

After the main study inclusion criteria were established and sample size calculations were complete, recruitment began. Mothers were recruited at prenatal WIC clinics in LA and at birth hospitals in Miami. Due to differences in the point of recruitment, the enrollment process varied slightly between sites.

In LA, the recruitment process began at a set of prenatal clinics within the catchment zip codes. Mothers were either approached by clinic staff or by recruiters directly. Interested mothers were told about the study using a scripted format and an eligibility screener was conducted. Interested mothers completed the screener and then, if eligible, the consent process was initiated at the clinic. The recruiters also secured consent at a later date if interested mothers felt that they could not immediately grant consent for their participation. In Miami, the recruitment process began at the well-baby unit of the recruitment hospitals soon after the mothers delivered their babies. Recruitment was conducted using a two-step process. The recruiters first approached mothers in the well-baby unit, with information about the study delivered using a scripted format. An eligibility screener was completed by all interested mothers. Within two weeks, eligible mothers received a follow-up visit by the recruiter at the homes of the mothers to complete informed consent and enrollment in the study.

Blinded randomization of the consenting participants was performed within each site (neither the site staff nor the participants knew the group assignment at enrollment) via a centralized, computer-generated process at the PCC. Assignments to either the intervention or comparison group were made on a weekly basis for enrolled participants. After randomization, the intervention site teams received assignments from the PCC and communicated them to the mothers.

To protect the rights of the research participants, mothers were asked for consent not only at the initial enrollment but again annually before each assessment visit. Separate consents were received for additional qualitative data collection on subgroups, including focus groups and case study interviews. Human subjects reviews were conducted by the Institutional Review Boards at CDC, at RTI, at UCLA, at UM, and at Western IRB for the time between 2005 and 2008 when UM contracted with them to conduct human subjects protection reviews.

### Participant characteristics

The final participant characteristics for the 574 mothers who completed the baseline assessment appear in Table 1. Statistical comparisons of each sociodemographic characteristic across the two sites were conducted in order to identify site differences. *T*-tests were used to contrast the continuous sociodemographic items (e.g., maternal age) and Chi-square statistics were used to compare distributions across categorical demographic variables. The sites differed significantly on a number of maternal characteristics, including age, education, marital status, cohabitation, race/ethnicity, and employment. The participant groups were similar for factors that reflected the recruitment criteria, including household income and the proportion of mothers speaking English in the home. The two groups were also similar in the proportion of male children, mothers living in public housing, and for indicators of the mother’s household composition.

**Table 1:** Baseline socio-demographics for mothers in the Miami and LA samples and the combined sample, by randomization group

	**Both sites (n = 574)**	**Miami (n = 289)**	**UCLA (n = 285)**	Test of Difference*
	**%**	**%**	**%**	**p-value**
Maternal Age < 23	49.3%	57.1%	41.4%	.0002
Child Female	49.9%	53.2%	46.8%	.7718
Maternal Education				
Less than HS diploma	23.9%	27.7%	20.0%	<.0001
HS diploma/GED	59.4%	61.9%	56.8%	
Voc. Tech/Associate's Degree	12.4%	9.0%	15.8%	
College degree or more	4.4%	1.4%	7.4%	
Maternal Marital Status				
Married	22.1%	16.6%	27.7%	0.0191
Separated	4.9%	4.8%	4.9%	
Divorced	2.1%	1.7%	2.5%	
Widowed	0.5%	0.4%	0.7%	
Never Married	70.4%	76.5%	64.2%	
Live with a Husband/Partner	40.2%	33.2%	47.4%	0.0005
Race/Ethnicity (multiple selections allowed)				
Non-Hispanic Black	57.1%	69.2%	44.9%	<.0001
Non-Hispanic White	3.8%	1.0%	6.7%	0.0005
Hispanic	24.9%	9.0%	41.1%	<.0001
Haitian	8.5%	17.0%	0.0%	<.0001
Other	5.8%	3.9%	7.9%	0.0446
Employment				
Working full-time	10.3%	11.8%	8.8%	0.0004
Working part-time	15.5%	9.7%	21.4%	
Not working	74.2%	78.6%	69.8%	
Household Income				
<$20,000	50.2%	52.6%	47.7%	0.5726
$20,000-29,999	19.5%	17.3%	21.8%	
$30,000-39,999	10.5%	9.7%	11.2%	
$40,000-49,000	6.6%	6.9%	6.3%	
$50,000+	4.9%	4.2%	5.6%	
Missing (imputed for analysis)	8.4%	9.3%	7.4%	
Primary Language Spoken at Home				
English	98.3%	98.3%	98.3%	0.9823
Non-English Language	42.3%	34.6%	50.2%	0.0001
Spanish	28.4%	13.5%	43.5%	<.0001
Creole	10.8%	20.8%	0.7%	<.0001
Other	5.8%	3.9%	7.9%	0.0446
English Spoken Most in Home	79.8%	85.1%	74.4%	0.0014
Home Ownership				
Owned	22.5%	29.2%	15.7%	<.0001
Rented	75.9%	69.8%	82.2%	
Occupied without rent payment	1.6%	1.0%	2.1%	
Lives in public housing	12.6%	14.5%	10.7%	0.1778
Household Composition		Mean		p-value
Number of adults living in home^	1.5	1.6	1.5	0.5781
Number of children living in home+	1.5	1.5	1.4	0.7054

* As indicated by Chi-square or *t*-test statistic, as appropriate.

^Excluding *Legacy* mother, top-coded at 6.

+ Excluding *Legacy* child.

Statistical comparisons of intervention and comparison groups were conducted within sites to identify any group differences that may have remained after group randomization. As depicted in Table 2, randomization of eligible participants to each of the two randomization groups within each site resulted in equivalence of groups across each measured sociodemographic characteristic.

**Table 2:** Baseline socio-demographics for mothers in the Miami and LA samples and the combined sample, by randomization group

	**Miami (n=289)**		**LA (n=285)**	
	**%**		**%**	
	**Interv.**	**Comp.**	**Interv.**	**Comp.**
Maternal Age < 23	55.5%	59.5%	40.6%	42.5%
Child Gender				
Male	47.4%	49.1%	56.5%	46.1%
Female	52.6%	50.9%	43.5%	53.9%
Education				
Less than HS diploma	29.5%	25.0%	21.8%	17.5%
HS diploma or more	70.5%	75.0%	78.2%	82.5%
Marital Status				
Married	14.5%	16.4%	26.7%	27.5%
Not Currently Married	85.6%	83.6%	73.3%	72.5%
Live with a Husband/Partner	33.7%	31.0%	47.8%	45.3%
Race/Ethnicity (multiple selections allowed)				
Non-Hispanic Black	65.9%	74.1%	43.6%	46.7%
Non-Hispanic White	1.7%	0.0%	5.5%	8.3%
Hispanic	9.8%	7.7%	43.6%	37.5%
Haitian	18.5%	14.7%	1.2%	3.3%
Other	4.1%	3.5%	6.4%	4.6%
Employment				
Working full- or part-time	21.4%	21.6%	28.5%	32.5%
Not working	78.6%	78.5%	71.5%	67.5%
Household Income				
<$20,000	53.8%	50.9%	49.7%	45.0%
>= $20,000	38.7%	37.1%	42.2%	48.3%
Missing	7.5%	12.1%	7.9%	6.7%
Languages Spoken at Home				
Non-English language	34.7%	34.5%	50.9%	49.2%
	Mean		Mean	
Household Composition				
Number of adults living in home*	2.6	2.6	2.5	2.5
Number of children living in home^	1.4	1.5	1.4	1.5

* Excluding *Legacy* mother, top-coded at 6

^ Excluding *Legacy* child

### Retention of subjects

Activities intended to maximize retention were implemented in both the intervention and assessment settings. Once a mothers was randomized to the intervention group, mothers received varying amounts of the intervention, depending on their own compliance. Mothers who ceased intervention participation were encouraged to rejoin the intervention at any time. Mothers who could not complete an assessment were invited for all subsequent assessments unless the mother refused participation. Whenever possible, an exit interview was completed for families electing to permanently disengage with the study before its completion. There were four reasons for permanently terminating the affiliation of a mother with the *Legacy* study. Enrolled participant mothers were dropped from the study if they 1) permanently refused participation, 2) permanently moved out of the catchment area, 3) were abusive within the group setting (e.g., threatened another participant), or in the case of 4 mother's or child’s death.

A number of specific study-related activities focused on maximizing retention in both the intervention and assessment settings. Our retention efforts began with an investment in continuous participant tracking. Each site employed staff members whose primary responsibility was to track and maintain contact with the participants. Tracking information included residential addresses, telephone numbers, alternate contact names and contact information, and preferred method of contact. These data were maintained electronically so that they could be viewed real-time by staff at both the intervention and assessment offices.

Incentives were also used to compensate participants for their time and efforts as well as to encourage study participation (Table 3). Although families in the intervention group were in more frequent contact with the *Legacy* staff than those in the comparison group (weekly vs. three times per year), efforts were made to minimize differential loss. Local staff kept in touch with the comparison families between assessment points through phone calls and mailings. Shortly after an assessment took place, written feedback was mailed to families. If the results of an assessment indicated a need for further services, mothers were provided with information on available services and referrals to outside early intervention services were facilitated.

**Table 3:** **Incentives used in the*Legacy for Children***^***TM***^**study**

			**University of Miami**			**UCLA**		
**Group**	**Periodicity**	**Purpose**	**Staff**	**Location/mode**	**Incentive**	**Staff**	**Location/mode**	**Incentive**
Comparison	Every six weeks	Maintenance contact	Trackers	Phone call	-			
	Annually	Holiday gift	Trackers	mail	$20			
Intervention	Prenatal – 36 m Weekly blocks	Group meetings				Int staff	Site	Meal/Child care ($25)
	Prenatal – 36 m After blocks	Home visits				Int staff	Home/site	Gifts/Materials
	Prenatal – 36 m before blocks	Session schedule reminder				Int staff	Mail	-
	0-60 m Weekly	Group meetings	Intervention staff (Int staff)	Site	Diapers/toys Child care ($20), small gifts			
	0-60 m monthly	Group meetings	Int staff	Site	$5 per attended visit			
	0-60 m yearly	Personal follow- up visits	Int staff	Home/site	tape			
	Annually	Holiday gift	Int staff	Site	$20			
	Annually	Parent Satisfaction	Assessors	Phone	$50	RTI	Phone	$50
Intervention subsample	Periodically	Focus group	Assessors	Site	$50	RTI	Site	$50
	Annually	Case studies	Assessors	Site	$50	RTI	Site	$50
Intervention and Comparison	Annually/ December	Holiday gift	Int staff/Trackers	Mail	$ ≤ 20			
	Annually	Mother birthday greeting	Int staff/ Trackers	Mail	-	Int staff	Mail	-
	quarterly	For complete assessments				Int staff	Mail	$25
	6 m	Assessment	Assessors	Assess office	$100	Assessors	Assess office	$100
	9 m	FUI	Trackers	Phone/home	$10	Trackers	Phone/home	$10
	9 m	FUI						
	12 m	Birthday greeting	Int staff/ Trackers	Mail	-	Int staff	Mail	-
	12 m	Assessment	Assessors	Assess office	$100	Assessors	Assess office	$100
	12 m	Assessment Home	Assessors	Home	$50	Assessors	Home	$50
	15 m	FUI	Trackers	Phone/home	$10	Trackers	Phone/home	$10
	15 m	FUI						
Intervention and Comparison	21 m	FUI	Trackers	Phone/home	$10	Trackers	Phone/home	$10
	21 m	FUI						
	24 m	Birthday greeting	Int staff/Trackers	Mail	-	Int staff	Mail	-
	24 m	Assessment	Assessors	Assess office	$100	Assessors	Assess office	$100
	~ > 24 m	Finger stick	Phlebotomist	Home	$50	Phlebotomist	Home	$50
	27 m	FUI	Trackers	Phone/home	$10	Trackers	Phone/home	$10
	33 m	FUI	Trackers	Phone/home	$10	Trackers	Phone/home	$10
	33 m	FUI						$10
	36 m	Birthday greeting	Int staff/Trackers	Mail	-	Int staff	Mail	-
	36 m	Assessment	Assessors	Assess office	$100	Assessors	Assess office	$100
	39 m	FUI	Trackers	Phone/home	$10	Trackers	Phone/home	$10
	42 m	FUI	Trackers	Phone/home	$10	Trackers	Phone/home	$10
	45 m	FUI	Trackers	Phone/home	$10	Trackers	Phone/home	$10
	45 m	FUI						
	48 m	Birthday greeting	Int staff/Trackers	Mail	-	Int staff	Mail	-
	48 m	Assessment	Assessors	Assess office	$100	Assessors	Assess office	$100
	48 m	Assessment Home visit	Assessors	Home	$50	Assessors	Home	$50
	51 m	FUI	Trackers	Phone/home	$10	Trackers	Phone/home	$10
	54 m	FUI	Trackers	Phone/home	$10	Trackers	Phone/home	$10
	57 m	FUI	Trackers	Phone/home	$10			
	57 m	FUI				Trackers	Phone/home	$10
	60 m	Birthday greeting	Int staff/Trackers	Mail	-	Int staff	Mail	-
	60 m	Assessment	Assessors	Assess office	$100	Assessors	Assess office	$100
Intervention and Comparison subsample	Pregnancy	Fetal monitoring				Specialist	Site	$50
							

Another key factor to facilitate mothers’ participation in the assessment and intervention component was overcoming common barriers to attendance. Both sites provided van transportation to assessment and intervention activities to any mother who wanted it. To maximize mothers’ ability to attend group meetings, van scheduling accommodated parents to the extent that was possible. In Miami, the decision was made to conduct meetings during the week. Initially, pilot Miami groups were conducted during the day but, as mothers returned to full-time employment, evening groups were added. In LA, all group meetings were initially conducted on the weekends, but experience with the pilot study showed that some weekday groups were necessary.

When a family moved out of the catchment area, the sites attempted to maintain communication with the mother so that future assessments could still be conducted. Mothers who returned to the area were invited back to participate in intervention and/or assessments during the course of the study. Reasons for non-retention (i.e., nonparticipation in any of the assessments) were documented on an individual basis.

### Analytic sample size and statistical power

#### Retention and sample size

**Figure 3:**
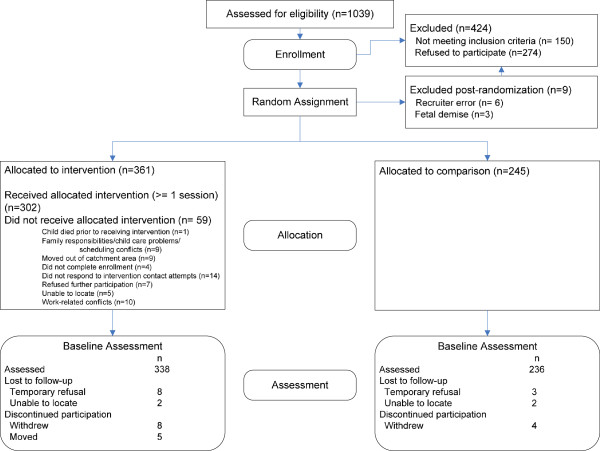
**CONSORT Flowchart for the Legacy for Children**^**TM**^** project.**

Table 4 reports the sample sizes for *Legacy* in terms of the number of completed assessments through age 5, with a full description of retention and participant loss by randomization group depicted in Figure 3. Although the *Legacy* impact assessment has been extended beyond age 5 and continues into school-age, the following discussion is constrained to the original impact assessment, conducted through age 5.

**Table 4:** **Sample size for*Legacy for Children***^***TM***^**, by site, year, and intervention status**

	**Los Angeles**			**Miami**		
**Year**	**Intervention**	**Comparison**	**% of Randomized Retained (n = 306)**	**Intervention**	**Comparison**	**% of Randomized Retained (n = 300)**
Baseline	165	120	93%	173	116	96%
1	138	100	78%	162	106	89%
2	136	92	75%	153	102	85%
3	127	79	67%	136	89	75%
4	124	78	66%	127	81	69%
5	117	73	62%	122	73	65%

The study began with a total of 338 intervention and 236 comparison baseline assessments, which represents 94% of the 361 mothers randomized to intervention and 96% of 245 mothers randomized to comparison. Baseline sample sizes were similar for the two sites. The mean assessment window for each assessment wave was within four to six weeks of the target date, from baseline to five years. Within each site, annual retention rates for each assessment wave were comparable across the two randomization groups. The sample sizes for each annual assessment are presented in Table 4 and culminate in Year 5 assessment rates of 64% across the two sites.

Attendance at group intervention sessions varied by site. In Miami, 90% of mothers attended at least one group session during the course of intervention provision. In LA, uptake was lower, with 78% of mothers attending at least one group session over the three years that intervention was delivered at the site.

### Measurement

In order to address the research questions of interest, three main categories of data were collected: a) process data on the intervention implementation and the mothers’ responses to the intervention; b) assessment data on mediating, moderating, and outcome variables; and c) cost data collected separately for intervention and research components. To develop and test the measurement protocol, complete data were collected for all participants in the pilot study, including process, outcome, and cost data.

#### Process measures

The process measures served several purposes. The first purpose was to determine the quality and fidelity of the intervention as implemented in each site for each of the key components included in the intervention design (e.g., parent groups, one-on-one visits, sense of community building efforts). In addition, the process measures provided feedback for formative purposes and ongoing assessment of program performance. This feedback was used to assist the intervention team in targeting training and technical assistance activities over the period of the study. Further, these data were used by the sites to monitor ongoing operations, in order to reduce their data burden and maintain investment in the study. Finally, the process measures contained qualitative information as a measure of intermediate outcomes (e.g., persistence of families in the program, engagement of participants) and key program components to assist in interpreting quantitative outcomes.

In short, the process measures were collected to evaluate whether the intervention conforms to the hypothesized model, whether the intervention was working and how it could be improved, the extent to which the intervention group actively participated in the program, and the causes and consequences of participant attrition. Thus, the process measures were used for both formative and summative evaluation.

#### Data sources

The process data consisted of time-based measures dependent on the mother’s random assignment to the intervention or comparison group. The process data were compiled from four data sources: (1) direct observation, (2) program record data, (3) data from program providers and (4) data from program participants.

##### Direct observation measures

A portion of all intervention sessions were directly observed by ethnographers. They followed selected groups at each of the sites throughout the *Legacy* project, but also observed other groups intermittently to document responses of different groups to similar intervention contents. The ethnographers described the *Legacy* meetings in detailed notes which were later coded and themed by trained and reliable coders.

##### Program record data

Program record data consisted of information about attendance and assessment rates, one-on-one visits, and information about contacts between *Legacy* staff and mothers. Forms were used to record information regarding one-on-one visits as well as individual contacts.

##### Data from program providers

Data were collected using interviews and questionnaires about how the group leaders perceived their success with the intervention as well as participants’ responses both on a group and individual level. At the completion of each group session, a parent group summary form was completed, documenting whether the session was completed as planned, as well as how mothers responded to the intervention and each other. A parent engagement form was completed every 10 weeks by the group leader for each mother. In addition to information regarding how many sessions each participant attended, the group leaders were asked for information regarding the perceived interest and participation of each mother in the main topics covered, the *Legacy* group, and their child. Also, the group leader rated the quality of the mother’s observed interaction with her child. These data are being used to calculate the dosage of the intervention received and to provide cross-validation of information provided by the mothers on the quantitative assessments.

##### Data from program participants

These data were collected through interviews, questionnaires, and focus groups. Focus groups of 10–12 randomly chosen participants were conducted annually by an evaluator from the PCC to explore the mothers’ perceptions and responses to the intervention curriculum, group leaders, and other participants. For each site, five mothers were selected for in-depth case studies and participated in annual interviews by an evaluator. The focus groups and case studies served to generate narratives about overall project experiences and gave respondents a chance to tell their story.

In addition, parent satisfaction surveys were conducted systematically for a subsample of participants, selecting those who actively participated (i.e., attended at least one session in the 6 months prior to the annual assessment time point). This interview solicited information about the mothers’ satisfaction with *Legacy*, the group leader, and the activities they may have taken part in. In addition, the interview solicited information regarding parental self-efficacy and relationships with other parents in the group.

The data gathered from the pilot study on process measures were used to refine the instruments in an iterative process. For example, when mothers in focus groups as well as in some of the group sessions discussed that they felt that being in *Legacy* was a unique experience, unlike any other group setting, a question was added to the satisfaction survey to learn whether this perception was shared by the majority of mothers or not.

#### Demographic, mediating, and outcome measures

The quantitative assessments were selected to measure the three constructs of interest: child development, parent/family characteristics, and parental sense of community. These constructs will be used to determine the degree to which the *Legacy* intervention influences child development and to analyze the paths of those impacts. The outcome domains for child development include cognitive development, language development, socio-emotional functioning, behavioral functioning, and health. In addition, data were collected on other parent and family domains that were expected to be important as either control variables or as mediators. These consisted of demographics and family background, self-efficacy, parental responsibility, parental investment, devotion of time and energy, parenting behavior (including guidance of behavioral and emotional regulation and facilitation of cognitive development), and quality of the mother-child relationship. The parental sense of community domains included support/resources/memberships, community/peer relations, satisfaction of needs (life and self), and psychological sense of community.

#### Data sources

Assessments were administered at baseline, when the child reached 6 and 12 months, and then annually until the child reached 5 years of age. The baseline assessment was designed to measure maternal characteristics, attitudes, and knowledge before the beginning of the *Legacy* intervention. The intervention began prenatally in LA and postnatally in Miami; thus, the baseline was administered prenatally (before intervention attendance was permitted) in LA and when the infants were 4–8 weeks old in Miami.

These assessments were conducted by assessment staff from the PCC who were blind to the intervention assignment. As an additional precaution, assessments took place in a lab that was in a separate building from the intervention, and contact between assessment and intervention staff was limited.

The assessment batteries consisted of questionnaires, direct assessments, and some videotaped observations of mother and child. Most of the measures were standardized and had been previously used in similar research projects, but some questionnaires were developed or adapted for *Legacy*. The majority of the measures were administered in person using a computer assisted personal interviewing procedure; however, some sensitive questions, e.g., regarding alcohol and drug use were completed by the mothers alone, using an audio-enhanced computer-assisted self-completed interviewing process. For a complete list of measures by time point, see Table 5.

**Table 5:** **Domain, constructs and measures by assessment time point in the*Legacy for Children***^***TM***^**Study**

**Domain**	**Construct**	**Measures**	**B**	**6**	**12**	**24**	**36**	**48**	**60**
Maternal Constructs									
Mediating Variables									
*Self Efficacy*			X	X	X	X	X	X	X
	*Parental Self Efficacy*		X	X	X		X		X
		*Components of Maternal Efficacy*[63,64]	X	X	X		X		X
		*Competence Subscale of PSI*[65]	X	X	X		X		X
		*Self-Efficacy Scale*[66]	X	X	X		X		X
	*Knowledge and Expectations*		X	X	X	X		X	X
		*Future Expectations *[67]		X				X	X
		*Knowledge of Infant Development Inventory*[68]	X		X	X			
*Commitment, Satisfaction*				X	X	X	X	X	X
	*Commitment/Involvement*			X	X	X	X	X	X
		*Parental Commitment Scale*[69]		X	X	X	X	X	X
	*Parental Role Satisfaction*				X		X		X
		*Parenting Satisfaction Scale*[70]			X		X		X
*Emotional Well-Being*			X	X	X	X	X	X	X
	*Stress, Mental Health*		X	X	X	X	X	X	X
		*Parenting Stress Index-Life Stress*[65]	X		X		X		X
		*Parenting Stress Index-Short Form*[65]			X		X		X
		*General Life Satisfaction*[71]	X	X	X	X	X		X
		*SF Mental Health Index*[72]	X		X		X		X
		*CIDI Depression*[73]	X		X	X	X	X	X
	*Coping Skills*		X	X		X	X		X
		*Coping Resources Interview*[74]	X	X		X	X		X
*Sense of Community, Support, Connectedness*			X	X	X	X	X	X	X
	*Social Networks*		X	X		X	X		X
		*Duke-UNC Functional Social Support Questionnaire*[75]	X	X		X	X		X

In addition to the baseline and follow-up assessments, a family update interview (FUI) was administered by the tracking staff every six months of child age from 9 through 57 months. These interviews included questions concerning contact information, household composition, child care, and employment and services received and were conducted by telephone or in person.

##### Development of the assessment plan

The approach to selecting child and family measures was based on relevance to the intervention goals and specific research question. The assessments included all child outcome domains, maternal mediating domains, as well as demographics and family background variables. Whenever possible, measures for which standardization samples and norms matching the current sample were available, as well as documented reliability and validity with an internal consistency reliability (alpha coefficient) of at least .70, were selected. Whenever feasible, measures that were appropriate to the mothers’ expected reading and comprehension levels and their cultural backgrounds were selected.

Developmental change is rapid from birth to 5 years, the age ranged covered in the *Legacy* study. Therefore, many measures of child outcome focused on a relatively narrow age range. Thus, to measure a particular outcome at different ages, different outcome measures had to be selected for some domains (e.g., Brief Infant-Toddler Social & Emotional Assessment [65] for younger ages and Devereux Early Childhood Assessment [66] for older ages to assess social/emotional development). Additionally, to accommodate the possibility that some children might have developmental lags, particular attention was paid to the floor (and ceilings) of the selected measures. A major issue of concern was the burden of assessment on the participants. Where possible we chose shorter assessments, and selected the most feasible measures in terms of time and materials.

Even with these selection criteria, several measures that were tested with the pilot participant group were dropped or replaced for the main study. One major cause for changes in methods was the fact that while mothers were asked whether they planned to teach English to their child, in reality many mothers who were bilingual had children who were either bilingual or mono-lingual non-English speaking, particularly at the earlier ages (primarily Spanish). Therefore, measures were selected, that were suitable for bilingual children, and, when possible, allowed for administration in Spanish. Specifically, the Kaufmann Assessment Battery for Children [67] was selected to measure cognitive development because it can be administered bilingually. The Spanish version of the Preschool Language Scales [68] was also included for those children deemed dominant in Spanish (as attained by maternal interview on child’s language exposure and preference).

Other measures turned out to be too challenging for the participant group. For example, the child version of the Violence Exposure measure [69] was too complicated for many children and was dropped. The direct child assessments of cognition and language were alternated with language assessed at 2 and 4 years of age and cognition at 3 and 5 years of age and the Bracken Basic Concept [70] scale was dropped because the testing time was too long for the children.

#### Cost

A major issue in evaluating the effectiveness of any early intervention model is to understand the cost of the intervention. Detailed cost measures regarding each component of the study, including intervention, assessment, and operational costs, were collected to document costs, and estimate cost benefit and/or cost effectiveness analyses.

#### Data sources

Detailed records of expenditures were kept for each resource category of labor, materials, equipment, buildings and facilities, and miscellaneous, as well as records of donated items. These records were supplemented by staff cost and activities diaries; *Legacy* staff members who routinely perform three or more *Legacy* activities were required to complete time and activity diaries. Each diary detailed specific activities and the respective amounts of time spent on each *Legacy* activity. These diaries were collected semi-annually.

### Analytic plan

#### Statistical approach

As described in the measurement section, *Legacy* gathered a rich dataset that included quantitative assessments of mothers and their children, qualitative ethnographic and quantitative process data from the intervention sessions, and cost data related to the administration and evaluation of the intervention as a whole. Throughout the course of the intervention, these data were described and compared across groups by intervention site through the use of classic intent-to-treat (ITT) analytic methods. The general analytic plan is described below.

#### Descriptive statistics

Descriptive statistics will be used with *Legacy* data for at least three purposes: 1) to compare the mothers who consented to participate in *Legacy* to the entire screened sample; 2) to examine the initial comparability of the intervention and comparison groups, 3) to provide a rich description of the Legacy mothers and their children at each time point. For group comparisons, parametric statistics will be used where appropriate and non-parametric statistics will be used where the assumptions needed for parametric tests are not met. For continuous variables, *t*-tests will be used to examine group differences and for categorical data Chi-square statistics will be calculated. For preliminary descriptive group comparisons, simulations, the Bonferroni correction method, or a false discovery rate approach will be used to account for multiple comparisons within an assessment domain.

#### Analyses of outcome and mediating factors

In general, all intervention effects will be investigated using site- and time-point-stratified models first, using an ITT approach. Due to important implementation differences, site pooled models with site interaction included, will be considered carefully and used sparingly. The primary outcomes will be first examined using *t*-test and Chi-Square analyses to investigate individual outcomes as a function of *Legacy* group assignment. Multivariate statistical modeling will be used to address changes over time as well as the effects of mediating and moderating variables. Given the longitudinal nature of the data, trend analyses will be employed to test for linear and curvilinear relationships in the repeated measures over time. In the anticipated event that significant group differences are detected in maternal and child mediators and outcomes, multivariate linear and logistic regression techniques will be employed as a means of identifying effect modification and controlling for significant confounders. All regression modeling procedures will follow the general conceptual path of influence described in the *Legacy* Logic Model (Figure 2). Multiple ANOVA or regression models that account for correlation of outcomes within subjects will be employed to model the collective influence of intervention randomization on multiple measures of a single outcome domain (e.g., cognitive/language outcomes). In anticipation of an increasing amount of missing data over time, multiple imputation and nonresponse weighting will be used as alternative means of conducting ITT analyses within a general PROC MIXED regression framework.

A strength of the *Legacy* evaluation is a design in which many mediators and outcome variables are measured at multiple time points. This will allow a mixed model approach. In the first stage, growth curves are fit for individuals. Depending on the number of measurement, these curves may be linear, quadratic, etc. In the second stage, predictors such as intervention or comparison group assignment, demographic characteristics, and other predictors will be added and evaluated for their ability to predict slope and intercept parameters.

Further, structural equation modeling will be used, as appropriate, to estimate multiple and interrelated dependence relationships and to represent unobserved concepts in these relationships and account for the measurement error in the estimation process. This type of analysis will be used to fit measurement and structural models based on the *Legacy* Logic Model. Models will incorporate multiple paths and mediating relations; in short, the models will represent paths through which the *Legacy* intervention might have an effect. In addition, multi-group models will allow us to compare sites, demographic groups, and the randomization groups for equivalency. A variety of indices will be used to evaluate these models.

### Analysis of process data

#### Intervention exposure

Participation frequency in the intervention groups serves as a measure of intervention dosage, and is expected to be related to the impact of the intervention. Participation will be examined both as a continuous and categorical measure, with intervention mothers being categorized into low, medium, high and non-participant groups. However, because there are likely to be confounding factors, such as demographics and maternal characteristics, that affect likelihood of participation, propensity scores corresponding to predicted attendance will be calculated for all mothers in the *Legacy* program. These scores will be used in dose–response analyses of primary outcomes to match intervention mothers with comparison mothers predisposed to the same level of participation (had they been randomized to intervention), balancing factors related to mothers’ ability to participate. In addition, qualitative process data related to retention and attrition will be examined.

#### Qualitative data

Ethnographic field notes made up a significant portion of *Legacy’s* qualitative data. A first purpose of the process data throughout the intervention was to provide feedback to the intervention sites on maternal responses to the intervention. For this analysis, the ethnographers reviewed their notes for major themes and observations, which were then compiled into monthly reports and shared with the intervention sites. Organized around *Legacy’s* major themes, the reports provided a narrative around the development of a sense of community among participants, parental self-efficacy, program response, and group dynamics.

For summative purposes, all ethnographic field notes were then themed and coded by trained coders from the PCC. During the pilot phase, the coding system was revised to accommodate new categories and themes, suggested by additional analysis of the data and field notes. Other forms of secondary analyses include case studies of individual participants, following the progress of selected participants over time, group case studies, following specific groups over time, and analyses of participant engagement.

### Analysis of cost data

Cost analyses will include both simple descriptive analyses and cost-effectiveness analyses to assess the cost per unit of outcome achieved. To date, cost estimates have been generated by site, type of intervention research activity (e.g., parent group meetings, visits to the home, and transportation), and at the family level. If participation in *Legacy* suggests improved outcomes among intervention participants, key outcomes will be identified to assess the intervention costs for unit increases in outcomes. Cost-effectiveness analysis is limited, however, in that multiple outcomes cannot be combined into a single measure of effectiveness. To overcome this limitation, estimates of cost savings may be summed from the literature for a number of outcome changes observed during and after the *Legacy* intervention. These estimated cost savings (benefits) could be compared to the costs of intervening to help assess whether the benefits of *Legacy* justify the costs.

## Discussion

### Strengths and unique contributions

The societal costs of poor developmental outcomes, including the personal costs to the individual and their families, are substantial and include medical, education, child welfare, social services, juvenile crime, and productivity loss. A concerted effort from multiple sectors and multiple disciplines, including public health, is necessary to address these societal concerns.

The CDC, in collaboration with other Federal and private partners, developed *Legacy* as a public health model to engage parents and promote evidence-based strategies that may contribute to overall child well-being in families in poverty. The results of the thorough evaluation of intervention effects, process, and costs will inform public policy on early intervention, health and well-being and help to address health disparities issues in at-risk populations.

### Study limitations

The *Legacy* study was designed as a rigorous trial of the intervention model, maximizing the internal validity so that potential alternate explanations for the results could be ruled out. In doing so, there was a corresponding cost to the external validity of the results. This trade-off may limit the generalizability of findings in three ways. First, the study was only conducted in two sites and with specific populations. Because of the low number of non-Hispanic White participants (15% at LA and 1% at Miami), the results may have limited generalizability for this population. Second, as mentioned previously, participant loss rate within the intervention group was 30%. Although mechanisms were in place to minimize subject loss, and the final sample had sufficient power to conduct the major analyses, fine-tuned examination of all factors will not be possible even with this sizeable sample. The results will thus reflect the population represented by mothers and children who were willing and able to continue participation in the assessments. Third, although there may be different patterns of effects within the individual intervention groups, there was a practical need to allow a certain degree of movement across intervention groups. Examples for this need include schedule conflicts, such as a return to work, as well as the need to merge groups over time due to group attrition. Therefore, although of interest, it is neither practical nor appropriate to perform analyses that treat the individual intervention groups as primary units of analysis. Finally, because the intervention was varied and adapted for each site, and specifically allowed for further adaptation of the content to mothers’ responses and comprehension level, fidelity to the model was difficult to measure precisely. Results may therefore be specific to the implementations and adaptations of UCLA and UM; therefore site differences cannot be fully explained quantitatively.

## Abbreviations

ANOVA: Analysis of Variance; BSC: Building Sense of Community; CLATT: Creative Learning Activities for Time Together; FUN Club: Family Unity Network Club; ITT: Intent-to-treat; MST: Main Session Topic; PCC: Project Coordinating Center; PCTT: Parent Child Together Time; RCT: Randomized Controlled Trial; UCLA: University of California at Los Angeles; UM: University of Miami.

## Competing interests

The authors declare that they have no competing interests.

## Authors’ contributions

All authors contributed to the manuscript. RP contributed to intervention and study design, ME contributed to analysis plan development, and analysis, SFV contributed to study design, analysis plan, data management, and analysis, AHC contributed to assessment protocol and analysis plan, KG contributed to intervention and study design, LHB contributed to intervention design, JH contributed to intervention design, LFK contributed to intervention design, and DCS contributed to intervention design and process evaluation. All authors contributed to writing and revising the paper. All authors read and approved the final version of the manuscript.

## Disclaimer

The findings and conclusions in this report are those of the authors and do not necessarily represent the official position of the Centers for Disease Control and Prevention.

## Pre-publication history

The pre-publication history for this paper can be accessed here:

http://www.biomedcentral.com/1471-2458/12/691/prepub
